# Using model explanations to guide deep learning models towards consistent explanations for EHR data

**DOI:** 10.1038/s41598-022-24356-6

**Published:** 2022-11-18

**Authors:** Matthew Watson, Bashar Awwad Shiekh Hasan, Noura Al Moubayed

**Affiliations:** 1 Department of Computer Science, Durham University, Durham, UK; 2 Evergreen Life Ltd, Manchester, UK

**Keywords:** Computer science, Machine learning

## Abstract

It has been shown that identical deep learning (DL) architectures will produce distinct explanations when trained with different hyperparameters that are orthogonal to the task (e.g. random seed, training set order). In domains such as healthcare and finance, where transparency and explainability is paramount, this can be a significant barrier to DL adoption. In this study we present a further analysis of explanation (in)consistency on 6 tabular datasets/tasks, with a focus on Electronic Health Records data. We propose a novel deep learning ensemble architecture that trains its sub-models to produce consistent explanations, improving explanation consistency by as much as 315% (e.g. from 0.02433 to 0.1011 on MIMIC-IV), and on average by 124% (e.g. from 0.12282 to 0.4450 on the BCW dataset). We evaluate the effectiveness of our proposed technique and discuss the implications our results have for both industrial applications of DL and explainability as well as future methodological work.

## Introduction

Recent advances in machine learning (ML) have resulted in models achieving (or in some cases exceeding^1^) human accuracy, leading to applications in increasingly complex and critical scenarios. For example, ML has been applied to critical care in medicine^2^ and metastatis prediction^3^. In such applications there could be dire consequences should the ML models perform poorly and inconsistently in production. However, increasing accuracy is not the only goal of ML models in these scenarios. It is also imperative that users (both domain experts and end-users alike) are able to *trust*^4,5^ the model’s decision; this encompasses not only accuracy, but also the robustness and interpretability of model output^6^.

In tandem with advances in the accuracy of models, much work has also been produced on the robustness and explainability of ML models’ output. Advances in robustness have seen the development of models and techniques to detect and protect against adversarial attacks^7–9^, improve uncertainty quantification^10^ and improve the ability of models to generalise to unseen data^11^.

However, neural based machine learning models are still inherently black boxes, with the exact reasons behind a model’s decision being impossible to easily discern. This becomes more evident with the advent of increasingly large models^12^, risking mistrust being placed in ML from both domain experts and end-users alike^13,14^.

There is also rising concern over the gap between training distributions and test (real-world) distributions, and how this gap might affect the underlying causal structures of the data^15^. There’s also a significant lack of understanding around why and how models generalise^16^, especially with recent work showing that over-parameterised networks are absent of the classic U-shaped test error curve^17,18^. The prevalence of shortcut learning in many neural networks^19^ can be used alongside the presence of inconsistent explanations^8^ to strengthen the argument the neural networks are not learning causal features.

For example, despite widespread claims of success in applying ML to COVID-19 tasks^20^, many of these models succumb to numerous pitfalls such as making spurious correlations or being unable to generalise^21,22^. Furthermore, explainable machine learning has been used to show how state-of-the-art ML models are not robust to small changes in training hyperparameters^23^, with identical models producing significantly different explanations when hyperparameters such as the random seed and training set order are changed. This lack of explanation consistency is of concern as it could completely erode the trust placed in these models.

In this paper we explore this problem further, by investigating and proposing solutions to the inconsistency between models trained on medical and biological tabular data. We focus on these applications as it is in these sensitive situations that inconsistent models pose the most significant risks and barriers to the adoption of ML. These are also highly specialised areas of expertise where interpretation of model output can have significant influence and can also be directly challenged. We propose a novel ensemble architecture that takes advantage of explainability techniques during model training to produce an overall model that is more consistent than the sum of its parts. We evaluate the effectiveness of this new architecture on the same tabular datasets as our initial experiments, and compare our results to the current state of the art. Finally, we discuss how this technique could be used in practice and identify potential future directions.

## Methods

We use the following notation to describe models and their explanations. A machine learning model $m_i$ is passed an input *x* to produce an output *o*, such that $m_i(x) = o$. For classification tasks, the final prediction *p* of $m_i$ is then the class with the highest predicted probability ${\mathrm{arg\,max}}\;m_i(x) = p$. An explanation for input *x* on model $m_i$ is given as $E_{m_i}(x)$, with the importance value for a given feature $x_{j, k}$ given as $E_{m_i}(x_{j, k})$.

### Explanation ensembles

Explanation ensembles are a novel ensemble architecture that improves explanation consistency. It has been shown in previous studies on the consistency of model explanations that ensemble models improve the explanation consistency when compared to non-ensemble models^23^. Furthermore, it has been frequently shown that ensemble architectures out-perform non-ensemble models, reduce the risk of overfitting and perform more complex classification tasks than would be possible with a single model alone^24^. More complex architectures have been shown to be more robust, be less susceptible to adversarial attacks and allow for better uncertainty quantification^10,11,25^. In particular, hyperparameter ensemble models have recently been proposed, wherein the ensembles not only combine weight diversity, but also hyperparameter diversity^10^—however, despite improving in many areas upon baseline models, these models do not show any significant improvement in explanation consistency^8^.

However, we have combined a modified ensemble architecture with a unique training procedure to create a model that produces consistent explanations by considering explanations from a wide set of models trained with differing hyperparameters. The final model is encouraged to use only important features that are shared between every model trained, resulting in a fully trained model that produces consistent explanations.

The core idea of our new architecture is that each ensemble consists of *S* sub-models $e_1, \ldots, e_S$, each of which is trained with a different hyperparameter setup. Note that only the random seed or order of the training set should be changed; hyperparameters such as learning rate and hidden layer size should remain identical across all *S* sub-models. The *S* sub-models are trained in tandem, with the loss function designed to force each $e_i$ to learn to use similar features (this is described in more detail in Section “The explanation ensemble discriminator”). The final explanation ensemble model is then an average across all sub-models, such that $E(x) = \frac{\sum _S e_i(x)}{S}$. In this section, we explain the explanation ensemble architecture in more detail, including the training process and discriminator that allows the sub-models to learn similar features.

#### The explanation ensemble discriminator

The aim of an explanation ensemble is to make each of the *S* sub-models to learn to use a similar set of features, with this being achieved through the training of a discriminator *D*. If the *S* sub-models cover a wide range of hyperparameters, then we expect that they will cover a wide range of learned features (this is follows from the results of inconsistent explanations shown in^23^), and as such the final model will have learned to ignore a large set of noisy (i.e. spurious) features. These two models are trained in tandem, in a minimax two-player game: the goal of *D* is to learn how to discern between real and fake samples while the goal of *G* is to learn the features of the true data distribution in order to fool *D* into making incorrect classifications.

We propose to use a discriminator *D* in the training of the ensemble model, which is trained on the explanations from the ensemble sub-models; the purpose of this discriminator is to then classify which of the *S* sub-models the explanation originated from. The goal of the training of the *S* sub-models is then to modify their weights such that the generated explanations then fool *D* into making incorrect decisions (whilst still balancing the final accuracy of the sub-models too). The exact details of this training process are described in Section “Explanation ensemble training”.

**Figure 1:**
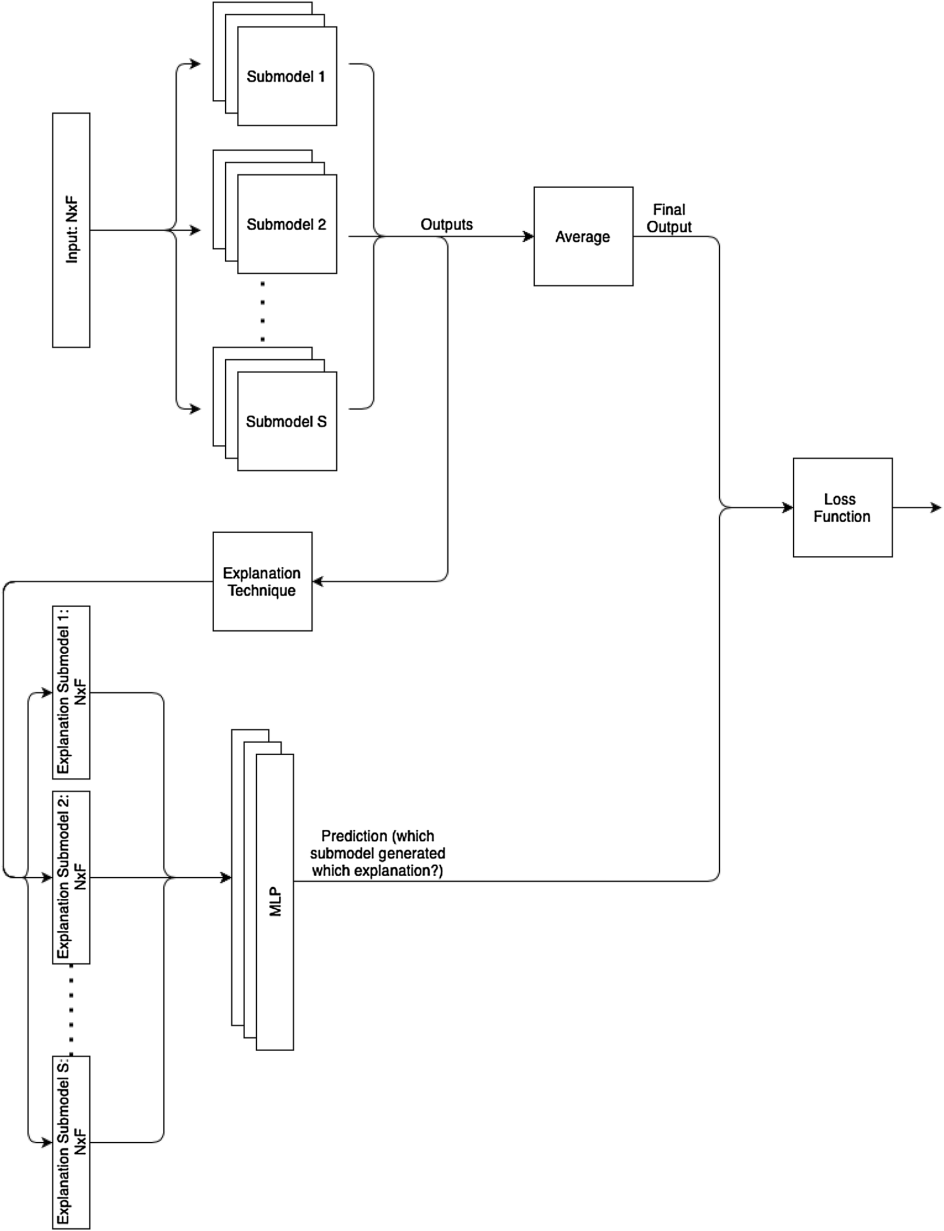
Diagram of our explanation ensemble architecture and data flow.

Our proposed discriminator *D* is a simple Multi-Layer Perceptron (MLP) with 1 hidden layer: there is an input layer (of the same size as the data samples), 1 hidden layer of size 32, a ReLU activation and finally an output layer (of size *S*, the number of sub-models). This discriminator joins *S* sub-models to create the whole explanation ensemble model, where each of the *S* sub-models can be of any architecture suited to the base task at hand (e.g. an MLP for classification or regression). Figure 1 shows an overview of our explanation ensemble architecture.

#### Explanation ensemble training

The training for explanation ensembles is the most important aspect of the model—there are a number of conflicting goals that it is aiming to achieve, and it is imperative that the training is setup in such a way that each of these goals can be achieved whilst also ensuring the model is easy to train. There are two objectives of the training process: (1) maximise model accuracy on the task at hand, and (2) minimise the difference between generated explanations of the *S* sub-models (i.e. maximise the error of *D*)—the resulting ensemble model should then have high performance/accuracy and, as the final feature importance values have been learnt across *S* different hyperparameters (and thus “averaged-out”), high(er) explanation consistency. Summarising these two objectives leads to the following loss function for the explanation ensemble

1 $$\begin{aligned} \text {loss} = \sum _i \texttt {CELoss}(m_i(x), y) - \beta \cdot \texttt {CELoss}(D(E_i(x)), i) \end{aligned}$$

where $\texttt {CELoss}(\cdot , \cdot )$ is cross-entropy loss, *y* are the ground truth labels for the training task and $\beta \in [0, \infty )$ is a hyperparameter for specifying the weight the discriminator plays during training. For all experiments in this paper, we set $\beta$ such that the two parts of the loss function have the same order of magnitude. This loss function requires that explanations are generated for each sub-model in each training epoch; any explanation technique (within the limits of the computational power available: many explanations techniques are too computationally intensive to make them viable options to be calculated across the whole training set *S* times each epoch) could be used here.

Equation (1) describes how the explanation ensemble model learns to fool the discriminator while minimising the classification (or regression, or other task-specific) loss. During an epoch where this loss function is used, only the weights of the *S* sub-models are updated—the discriminator remains the same. Thus, every *n* epochs *just* the discriminator *D* *alone* is trained (without back-propagating through the sub-models), allowing the discriminator to learn how to accurately classify which sub-model a given explanation was calculated from. Previous work on explanation consistency shows that, for many (if not most) tasks, this explanation classification task is easy for an ML model to learn to a high degree of accuracy^23^ (in fact, this is a direct result of the fact that ML models so far have shown low levels of explanation consistency) and so *D* is able to learn how to do so in a single epoch. To summarise, the general training process of an explanation ensemble is as follows:

1. For each $i \in [S]$ run $m_i(x)$ with the correct hyperparameters (i.e. training seed)
2. Calculate the explanations $E_i(x)$
3. If $e \mod n = 0$ update the discriminator *D* using the loss $\texttt {CELoss}(E_i(x), i)$, where *e* is the current epoch
4. Otherwise, update each of the *S* sub-models according to the loss function in Eq. (1)

  Training of our proposed architecture is inherently unstable; for instance, the loss of the discriminator is minimised if every feature in the data is given the same importance value—however, for this to be possible each of the sub-models must necessarily be outputting the same class, regardless of the input *x*. This leaves *n* as a hyperparameter that can be optimised (e.g. using a grid-search), though as an initial starting point $n=2$ has been found to result in stable training across all experiments.

### Explanation computation

To generate explanations for all of our models, we calculate the SHAP^26^ values across the whole dataset. As discussed in Section “Explanation ensembles”, SHAP is highly versatile and can be applied to any data modality; alternative feature attribution methods such as Grad-CAM^27^ and Information Bottleneck Attribution^28^ are restricted to certain data types. We follow the method detailed in^23^ to calculate the explanation consistency for these models.

### Alternative explanation consistency calculations

There are a number of other methods that can be used to measure the consistency of the model explanations^23^. One alternative is to approach the problem from an information theoretic background, using statistical distance measures to quantify the difference between the produced explanations. Being symmetric, smooth, and bounded Jensen-Shannon Divergence (JSD) is aptly suited to this task^23,29^, allowing the comparison between the probability distributions of the explanations for two models. The main disadvantage of this technique is that JSD is only defined for probability distributions, whereas we only have a finite number of samples for each model’s explanations. To alleviate this issue, we perform Kernel Density Estimation (KDE) on the explanations from a model to estimate the probability density function. For each dataset/task pair, we perform KDE on the explanations for each model (both baseline and explanation ensemble models). Then, for each pair of baseline models and each pair of explanation ensembles (for a given task), the JSD is calculated, with higher values indicating the two sets of explanations are dissimilar. This can be used to calculate the JSD consistency of the explanations

2 $$\begin{aligned} C_{JSD} = 1 - \frac{\sum _{(a,b)} J(a \ \parallel \ b)}{\alpha } \end{aligned}$$

where $J(a \ \parallel \ b)$ is the JSD between the explanations of model *a* and model *b*.

### Explanation quality metrics

To test the faithfulness of our explanations to the models (that is, to ensure that the explanations are accurately describing the changes in the model), we use three different explainability quality metrics: explanation sensitivity^30^, explanation infidelity^30^ and explanation accuracy^12^.

### Statistical hypothesis testing

As well as reporting the results for both performance and consistency we also investigate the statistical significance of our results, performing statistical hypothesis tests on both the model performance results and the explanation consistency results. Note that we cannot assume that our data (i.e. the performance metrics and explanation consistency) is normally distributed, and so parametric tests such as Student’s *t*-test are not viable. Similarly, we cannot assume that the distribution of the differences between the baseline ensembles and explanation ensembles are symmetric and so the Wilcoxon Signed Rank test would also be invalid. Instead, a non-parametric version of these tests must be used—specifically, we use the Mann-Whitney U test, setting the null hypothesis $H_0$ as the two distributions being equal.

We calculate and report the test statistic *U*, and the corresponding *p* value, for each dataset, comparing both the performance metric and the separability between the baseline ensembles and explanation ensembles. We perform the hypothesis tests at the $\alpha = 0.05$ significance level, meaning that the null hypothesis $H_0$ will be rejected if $p < 0.025$ (as we are using a two-sided version of the Mann-Whitney U test).

### Ablation study

Like any ensembling technique, explanation ensembles are more computationally expensive during inference time than traditional models, and that this may have an impact on their use in production environments^31^. It is also important for us to determine that all parts of our technique are critical to the end result, and that improved explanation consistency is not a result of a single part of the system. We test three post-training methods that attempt to address this issue: submodel averaging, random sub-model selection, and a combination of checkpoint and submodel averaging. Checkpoint averaging is a weight averaging technique that has been shown to lead to better model generalisation^32^. We perform checkpoint averaging (by taking the 10 most recent saved checkpoints at the end of training) on both the baseline models and the normal ensemble models, calculating the explanation consistency for these two techniques as detailed above. In submodel averaging, we create a single model by averaging the model weights of each of the *n* sub-models trained in the explanation ensemble—this results in just one model that will be much quicker to run at inference time. In random submodel choosing, we simply pick one of the sub-models of the explanation ensemble at random to use at inference time; with the intuition being that, as the model has still been trained to produce explanations similar to those of the other $n-1$ sub-models, it should still produce better explanations than traditionally trained models. We also combine the checkpoint averaging technique that we have tested on normal architectures with submodel averaging.

## Results

To thoroughly test the ability of our proposed explanation ensemble model to improve the consistency of the produced explanations, we train 6 different tasks on 4 distinct healthcare/biological datasets. First, we introduce the tasks and datasets, explain the motivation behind the inclusion of each dataset, then report the results of our experimentation.

### Datasets and base model architectures

In an effort to keep these initial experiments as simple and interpretable as possible, we limit ourselves to tabular datasets. Decisions based on tabular data are inherently easier to understand and explain—there are a (typically small) number of distinct features, and often these features will be well understood by domain experts. In contrast, features (and thus explanations) of more complex data modalities are harder to define. For example, in an image each individual pixel is a feature and yet humans (and indeed many ML models) will utilise superpixels (groups of pixels) when making decisions. This makes explanations on these data types more difficult to analyse. It also introduces difficulties when comparing the explanations of two different samples—in tabular data, feature importance values can be directly compared, whereas this comparison is difficult to accurately define as the features between most other data modalities are not necessarily aligned. For these reasons, we limit our evaluations in this study to the effectiveness of our proposed methods on tabular data, and leave investigations on other data types to future work.

Deep learning models are being increasingly used to analyse Electronic Health Record (EHR) datasets for the prediction of mortality, phenotyping, de-identification and other related tasks^33^. Further examples of tabular dataset come from genome analysis, on tasks such as pattern identification and kingdom classification^34^. The application of ML to both of these areas also rely heavily on model interpretability, and the trust of domain experts (e.g. clinicians and biologists)^6^, and so by extension consistent explanations from models are imperative. The purpose of this paper is to investigate the (in)consistency of explanations produced by models on these datasets, and inspect whether our proposed explanation ensemble architecture improves upon the consistency. Therefore, for each dataset, we re-implement a state-of-the-art neural network for the given dataset and use this model as the base for our consistency experimentation. This results in a *base model architecture* for each dataset/task which forms the basis of our experimentation. We then take these base model architectures and use them as sub-models to train a *normal ensemble architecture*, as well as our proposed *Deep Explanation Ensemble (DEE) architecture*. This allows comparison of our proposed network with both a standard baseline and an ensemble baseline. A summary of the datasets, tasks and baseline models (and hence ensemble sub-models) used can be found in Table 1.

#### EHR datasets

We use three different EHR datasets. The Breast Cancer Wisconsin (BCW) dataset^35^ is a small, classical ML dataset that has been used frequently as a baseline test for the performance of ML models on healthcare data. Each entry in the BCW dataset consists of a set of features extracted from a digitized image of a fine needle aspirate of a breast mass, with the features describing: radius, texture, perimeter, area, smoothness, compactness, concavity, concave points, symmetry and fractal dimension. The aim of the task is to train a classification model to predict which tumors are malignant. Following the results of^36^, we use a small Multi Layer Percetpron (MLP) for this classification problem. The MLP consists of an input layer, a hidden layer of size 40, a second hidden layer of size 15 and then the output layer; we use the ReLU activation function, with LogSoftmax being used on the output of the final layer. The model is trained over 14 epochs, with a learning rate of 0.001, batch size of 64, Negative Log Likelihood (NLL) loss and the Adam optimiser.

The second EHR dataset we use is KAIMRC: a private EHR dataset collected from King Abdulaziz Medical City located in the central and western regions of Saudi Arabia^37^. The dataset spans 2016 to 2018, and includes patient demographics (e.g. age and Body Mass Index), lab results (e.g. cholesterol levels) and vital signs during this period. For a detailed description of the features included in the dataset, and their clinical relevance, we refer the reader to^37^. The dataset was collected to aid the development of ML models for diabetes prediction. We use this dataset for two separate, but related, tasks. (1) We train a classifier to predict patients with elevated HbA1c levels using longitudinal data, and (2) We train a regression model to predict HbA1c levels. The KAIMRC classification task is similar to the BCW task in that it is a binary classification problem, but the KAIMRC dataset is much larger and more complex than BCW and thus has been chosen to evaluate our proposed explanation ensemble models on real-world datasets. Similarly, the KAIMRC regression task is used to verify our proposed methods work on regression as well as classification. We follow the methods presented in^37^ to create our MLP models. The KAIMRC classification MLP uses 3 hidden layers of sizes 48, 48, and 24 respectively, using ReLU activation functions after each hidden layer and Sigmoid on the output. Mean-squared error (MSE) was used for the loss function and the Adam optimiser was used. The KAIMRC regression model follows the same general structure, with dropout with probabilities 0.2 and 0.1 after the first and second hidden layers respectively.

The final EHR dataset used is MIMIC-IV^38^. MIMIC-IV is a large, freely-available medical dataset collected from the critical care unit of Beth Israel Deaconess Medical Center from 2008 to 2019. MIMIC-IV contains patient information (e.g. age, weight, height, comorbidities), lab events (e.g. cholesterol, creatinine, bilirubin, HbA1c levels), vital signs and medication prescribed of 383,220 patients; for a more detailed description of the dataset, we direct the interested reader to^38^. MIMIC-IV is a time-series dataset and as such each record (e.g. patient) will have a different number of features, and the exact features present for each record will vary. We follow the flexible-ehr framework^39^ to train a model for mortality prediction. flexible-ehr consists of an embedding layer (embedding the input to a layer of size 32) followed by a Long Short-Term Memory (LSTM) module (with a hidden dimension of size 256), which is then passed into an MLP (with 4 hidden layers of sizes 32, 64, 128, and 256)^40^. We follow exactly the setup and hyperparameters suggested in the original paper, and report these in Table 1. This dataset and model architecture not only allows us to both test the ability of our explanation ensembles to perform on very large-scale datasets, but also the effectiveness of explanation ensembles on complex sub-model architectures; all other experiments in this paper use MLPs of varying layouts, whereas flexible-ehr is a much more complex architecture consisting of an embedding layer, LSTM and MLP.

#### Genomic datasets

We utilise one genomic dataset for two different tasks. The codon usage dataset^41^ consists of the usage frequency of 64 codons for more than 13,000 organisms. We follow the methods presented in^41^ to train two different models; one to classify the organisms kingdom (from 5 distinct classes), and the other to classify the DNA type of the organisms (from 3 possible classes). We perform the same data pre-processing (removing organisms with less than 1000 codons and those with DNA types in categories 2 or higher), resulting in 12,964 samples in the final dataset. As per their methods, both MLPs consist of a single hidden layer with 9 neurons. The purpose of evaluation our methods on these two tasks is to evaluate the performance of our explanation ensembles on multi-class classification problems (whereas previous classification-based experiments are exclusively binary classification problems).

**Table 1:** Summary of the dataset and tasks used to evaluate Deep Explanation Ensembles alongside baseline model and training hyperparameters.

Dataset name	Dataset descriptors				Baseline model hyperparameters		Baseline training hyperparameters		
	Task	Num. samples	Num. features	Num. classes	Model architecture	Num. hidden layers	Num. epochs	Learning rate	Batch size
Breast cancer Wisconsin	Binary classification	569	10	2	MLP	2	14	0.001	64
KAIMRC	Binary classification	18,844	24	2	MLP	3	14	0.001	32
KAIMRC	Regression	18,844	24	N/A	MLP	3	14	0.001	32
MIMIC-IV	Binary classification	383,220	N/A	2	flexible-ehr	4	20	0.0005	128
Codon usage (Kingdom)	Multi-class classification	12,964	64	5	MLP	1	16	0.0001	32
Codon usage (DNA)	Multi-class classification	12,964	64	3	MLP	1	20	0.0001	32

Note that MIMI-IV is a time-series dataset and so each entry will have different numbers of features, and the KAIMRC (Regression) task has no target class as it is a regression problem.

### Model performance results

We train multiple versions of each baseline model and inspect how changing their training hyperparameters affects model performance and explanation consistency. For each training task we systematically change the training hyperparameters, changing only one hyperparameter at a time, in order to isolate the affect of each change. We investigate both changing the random seed and training set order. For each task we train 10 models with the same random seed but different training set orders, and another 10 models with different random seeds but the same training set order. Each model is given the same train/test split—it is only the order the training set is passed to the model that is changed.

Traditional ensemble models are also trained on each classification task. Each ensemble consists of 10 sub-models, using the same architectures described in Section “Datasets and base model architectures”. We compare and contrast the results of these models with those from the explanation ensembles in order to discern whether any changes in model performance/consistency originates from the use of the general ensemble architecture or our specific explanation-based architecture.

We also train our proposed deep explanation ensemble architecture with the baseline model architectures for each task used as the deep explanation ensemble sub-model as detailed in Section “Datasets and base model architectures”. We begin by using 10 sub-models per ensemble. As detailed in Section “Explanation ensembles”, the discriminator is trained on alternate epochs and with a low learning rate of 0.00001. Qualitative experiments show that $\beta$ should be set such that the discriminator loss is one order of magnitude less than that of the classification loss, and so $\beta = 0.1$. 10 explanation ensemble models are trained with different random seeds (but keeping the training set order the same) and 10 models are trained with different training set orders (but the same random seed). We repeat these experiments 3 times (with different seeds/orders) to allow for the calculation of standard error. We record the performance of the models and the consistency of the explanations, and compare the results with those from the base models. Similarly to our experiments on the normal ensembles and baseline models, we also test checkpoint averaging of explanation ensembles.

Tables 1, 2, 3 and 4 in the Supplementary material report the performance metrics and hyperparameters used for each individual baseline model trained, for the KAIMRC, BCW, Codon Usage and MIMIC-IV datasets respectively. We compare these results to the current state-of-the-art results for each dataset. The reason for this is twofold: (1) it ensures that when we compare the results of the explanation ensembles to the baseline models we can easily compare it to state of the art models, and (2) it will help to confirm that any explanation inconsistency present in the baseline models are not the result of improper training. Table 2 shows a summary of the performance of our baseline models, alongside the variation in performance when training hyperparameters are changed. It also highlights how all of our baseline models achieve equal (or near-equal) levels of performance compared with the current state of the art for their respective tasks.

**Table 2:** Summary of mean accuracy/$R^2$ (± standard deviation) for the baseline models when the seed and training set order is changed during training.

Dataset (Task)	Seed	Shuffle	Overall	SotA performance
KAIMRC (Classification)	82.576 ± 0.4668	83.381 ± 0.1811	83.12 ± 0.4779	83.22
KAIMRC (Regression)	0.5927 ± 0.0113	0.579 ± 0.0122	0.5858 ± 0.01326	n/a
BCW	92.185 ± 1.7315	91.5 ± 2.8319	91.843 ± 2.3172	99.04
Codon usage (Kingdom)	85.280 ± 1.8029	85.38 ± 1.1778	85.33 ± 1.4367	84.25
Codon usage (DNA)	99.268 ± 0.0950	99.166 ± 0.0921	99.217 ± 0.1033	99.15
MIMIC-IV	76.362 ± 2.5808	79.736 ± 1.8906	78.049 ± 2.7769	84.72

The state of the art (SotA) model performance is also reported to confirm the models are properly trained.

Similarly, we report the performance of our baseline “normal” ensemble models in the same way. Tables 5, 6 and 7 in the Supplementary Material list the accuracy of each individual model trained, and the hyperparameters used during training. A summary of the spread of performance of the baseline, normal ensemble models is shown in Table 3—by comparing this table with the results in Table 2, we can see that the normal ensemble models neither improve nor degrade performance when compared to our baselines and the current state of the arts.

**Table 3:** Summary of mean accuracy/$R^2$ (± standard deviation) for the normal ensemble models and explanation ensemble models (ours) when the seed and training set order is changed during training.

Model architecture	Dataset (Task)	Seed	Shuffle	Overall
Normal ensemble	KAIMRC (Classification)	83.244 ± 0.2367	83.212 ± 0.0920	83.228 ± 0.1702
	KAIMRC (Regression)	0.51 ± 0.01673	0.524 ± 0.03007	0.517 ± 0.02532
	BCW	77.890 ± 11.563	71.736 ± 11.466	74.813 ± 11.330
	Codon usage (Kingdom)	90.134 ± 1.6527	90.568 ± 1.2715	90.351 ± 1.4088
	Codon usage (DNA)	99.150 ± 0.2141	99.122 ± 0.2270	99.136 ± 0.2086
	MIMIC-IV	77.23 ± 0.7935	75.96 ± 0.6299	76.60 ± 0.7520
Explanation ensemble (Ours)	KAIMRC (Classification)	72.173 ± 0.3998	72.423 ± 0.6856	72.298 ± 0.5365
	KAIMRC (Regression)	0.5504 ± 0.0181	0.5408 ± 0.0243	0.5515 ± 0.0197
	BCW	87.824 ± 4.0860	86.783 ± 2.5061	87.361 ± 3.3173
	Codon usage (DNA)	98.032 ± 0.5420	97.6523 ± 0.5411	97.863 ± 0.5447
	Codon usage (Kingdom)	89.176 ± 1.1797	89.055 ± 1.0404	89.110 ± 1.0497
	MIMIC-IV	77.338 ± 0.000083667	77.32 ± 0.0001225	77.329 ± 0.0001370

**Figure 2:**
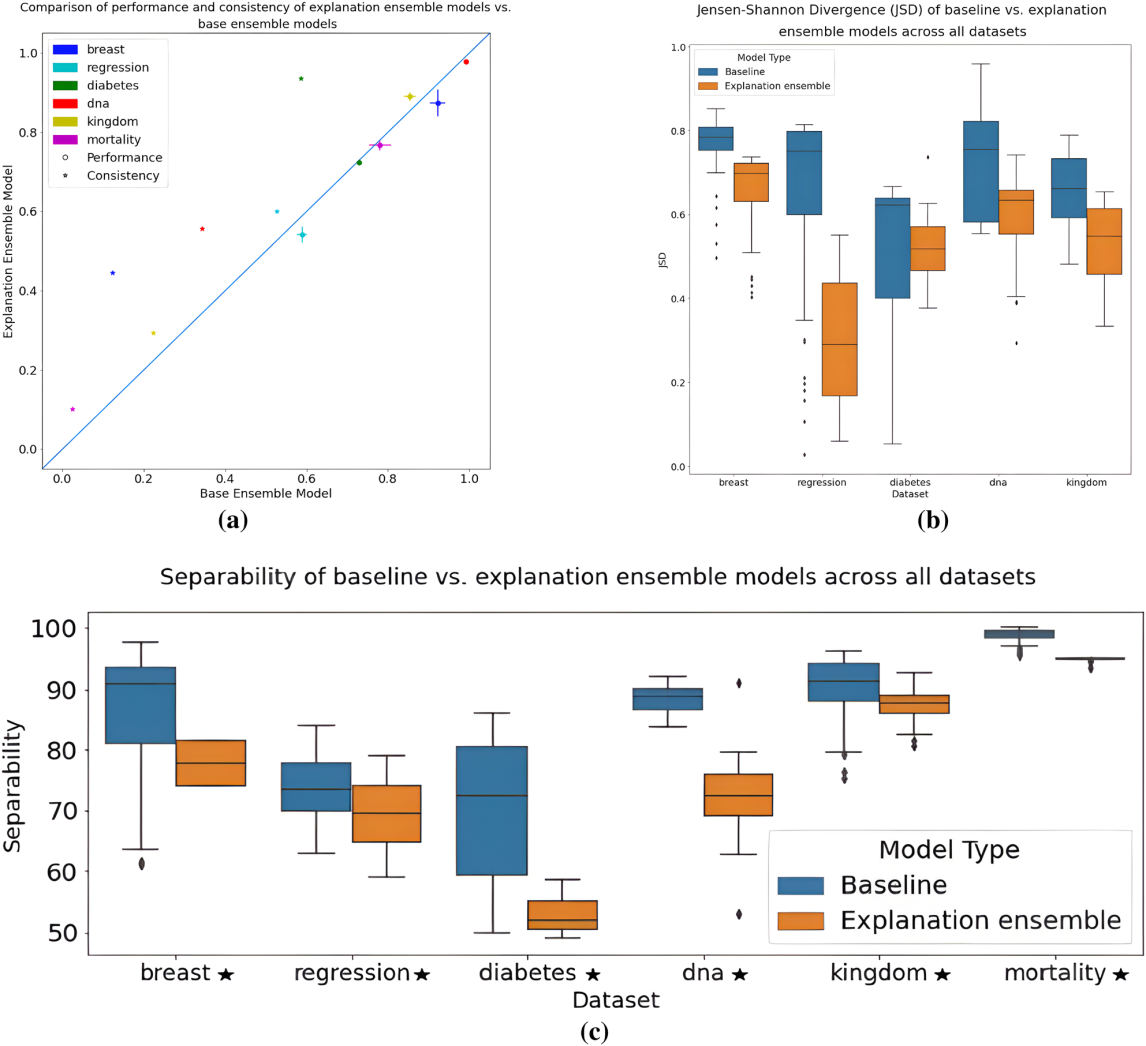
Figures comparing the explanation consistency *C* (**a**), JSD explanations consistency $C_{JSD}$ (**b**), and explanation separability (**c**) between baseline models and our proposed explanation ensembles across all tasks tested. Stars indicate datasets where the difference between the two architectures is statistically significant, following the results of a Mann-Whitney U test.

**Figure 3:**
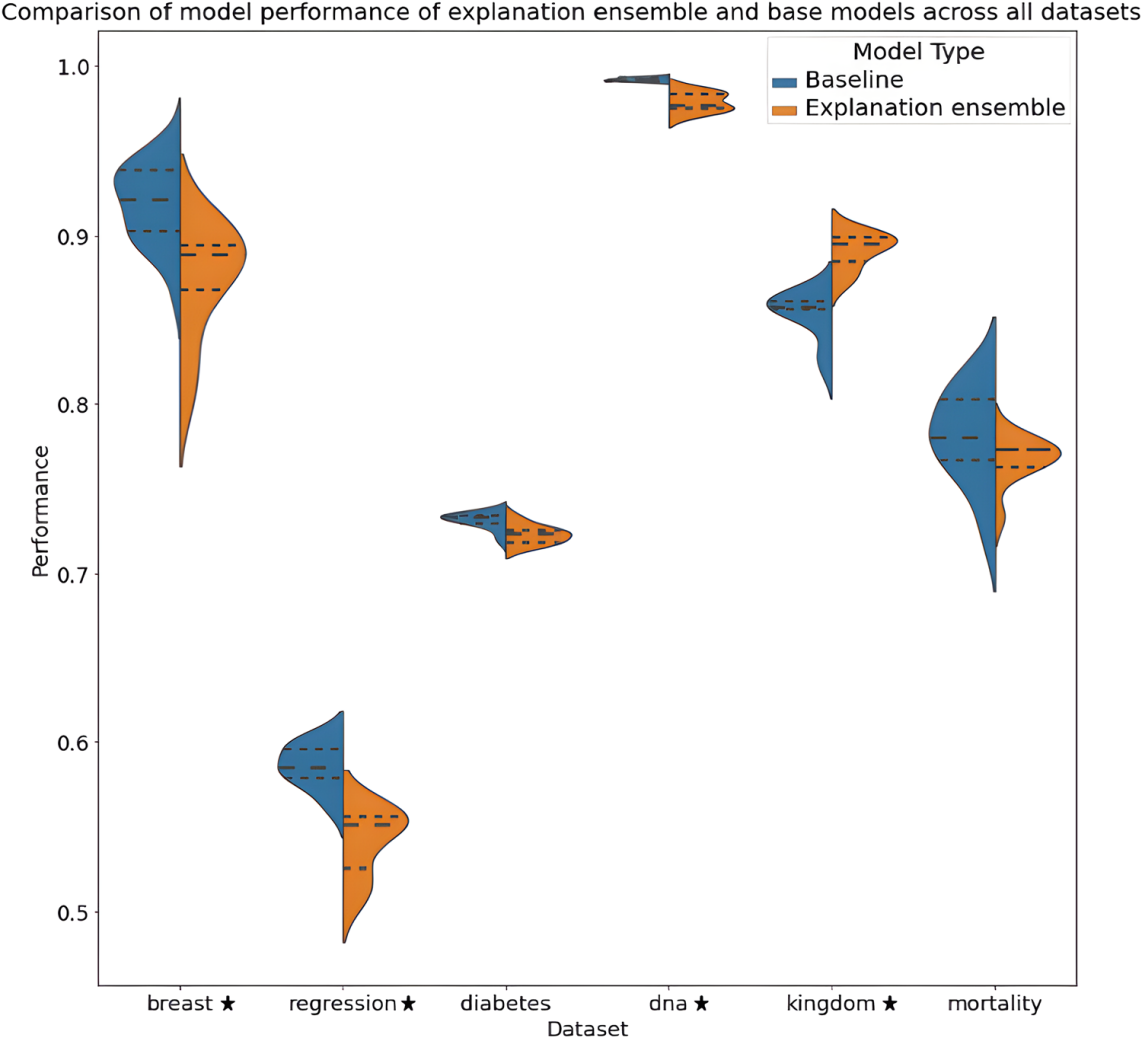
Violin plots showing distribution of model performance across all datasets, for both the base and explanation ensemble architectures. The dashed lines represent the 25th, 50th and 75h quantiles respectively. Performance for the Breast, DNA and Kingdom datasets is measured as $\frac{\text {accuracy}}{100}$, regression uses $\bar{R}^2$ and mortality AUROC. Stars denote datasets where there is a statistically significant difference in the two architectures, following the results of a Mann-Whitney U test.

The performance of the baseline models is compared against the performance of explanation ensemble models trained on the same task. Tables 8, 9, 10, and 11 in the Supplementary Material report the individual performance of our explanation ensembles, alongside the hyperparameters used during training. Table 3 summarises these results, and also shows the degree of variation when training hyperparameters are changed. This information is summarised in Figs. 2a and 3, which highlight the differences in spread and location of model performance when training hyperparameters are changed. For all datasets the mean explanation ensemble performance is always within a 10% range of the base model performance; although this does represent a slight decrease in model performance when explanation ensembles are used, we argue that this is only a slight decrease that would be worth the trade-off given that explanation consistency is significantly improved.

### Explanation consistency results

Table 4 reports the consistency, *C*^8^, on all tasks tested—*C* is calculated for all training variations and architectures. Table 4 shows that our proposed explanation ensemble architecture significantly improves the consistency of the produced explanations.

Table 4 also shows that the degree to which explanation consistency improves varies greatly on the dataset/task—for example, the Codon Usage Kingdom classification task sees an increase of only 0.07167 whereas the KAIMRC classification task sees and increase of 0.35417. We hypothesise that this is due both to differences in the dataset and differences in the baseline model (and thus also the explanation ensemble sub-models) architectures. The KAIMRC dataset consists of only 2 classes and 24 features, whereas the Codon Usage Kingdom classification task has 5 classes and 64 features; intuitively, one would expect it would be easier for the explanation ensemble models to learn consistent features for the smaller, simpler KAIMRC dataset than the Codon Usage dataset.

**Table 4:** Explanation Consistency (*C*) and JSD Explanation Consistency ($C_{JSD}$) for the baseline models and explanation ensembles across all tasks tested.

Dataset (Task)	Base mModel $\mathbf {C}$	Explanation ensemble $\mathbf {C}$	Base model $\mathbf {C_{JSD}}$	Explanation ensemble $\mathbf {C_{JSD}}$
BCW	0.12282	**0.4450 (262%)**	0.24682	**0.273065 (11%)**
Diabetes (Classification)	0.58550	**0.93697 (60%)**	0.51667	**0.543646 (5%)**
Diabetes (Regression)	0.52691	**0.600067 (13%)**	0.35389	**0.65568 (85%)**
Codon Usage (DNA)	0.34279	**0.5564 (62%)**	0.28114	**0.340558 (21%)**
Codon usage (Kingdom)	0.22220	**0.29387 (32%)**	0.34702	**0.39391 (14%)**
MIMIC-IV	0.02433	**0.10111 (315%)**	0.15518	**0.17912 (15%)**

The percentage increase from baseline *C* ($C_{JSD}$) to explanation ensemble *C* is shown in brackets.

Significant values are in [bold].

Figure 2c demonstrates the difference in spread of the mean separability, $S_{(a, b)} = 2 * | M_{(a, b)} - 0.5|$, between each individual training variation tested. This allows for a more fine-grained analysis of the explanation consistency than the high-level summary that explanation consistency *C* provides, noting that the higher the separability the “worse” the results. Figure 2c shows that the mean separability of explanation ensembles is lower than that of the baselines across all datasets, and that the separability is also spread across a lower range of values than both the baseline models and baseline ensemble models. These figures confirm that the discriminator portion of the explanation ensemble architecture is successfully encouraging each ensemble sub-model to learn similar features, and that this is in turn successfully forces models with different training hyperparameters to learn similar features.

As also reported in Table 4, we verify these explanation consistency results by also calculating the JSD consistency, $C_{JSD}$ (Eq. 2), for each dataset. These results conclusively confirm the results of the original consistency measure *C*, with the baseline models having low $C_{JSD}$ and explanation ensembles having higher $C_{JSD}$ values. Figure 2b showcases these difference in JSD values across the baseline and ensemble models—the similarity to Fig. 2c further confirms our results.

Table 5 reports the results of checkpoint averaging, submodel averaging, random submodel picking and checkpoint averaging followed by submodel averaging. The results are consistent across all architectures: neither checkpoint averaging, submodel averaging nor random submodel picking improves explanation consistency. When compared to the results of the baseline techniques in Table 4, explanation consistency decreases when these extra steps are added, confirming that our proposed method produces the best results. In the case of submodel and checkpoint averaging, we hypothesise that this is the result of the averaged model using noisy features from all of the models used in the averaging process, whereas our explanation ensemble technique is designed to instead encourage *all* models to learn to use similar features *before* the averaging takes place. Conversely, our explanation ensemble technique is not powerful enough to force each of the submodels to learn *exactly* the same set of features, with this explaining why using one of the trained explanation ensemble submodels (at random) doesn’t work as well; the averaging out of the (smaller than normal) set of noisy features across each of the submodels in the explanation ensemble plays a large part in the generation of consistent explanations.

**Table 5:** Explanation consistency, *C*, of checkpoint averaging, submodel averaging and random submodel picking on baseline models and both normal and explanation ensembles. CA-SA is checkpoint averaging followed by ensemble submodel averaging, CU the Codon Usage dataset, class. is classification and regr. regression.

		BCW	Diabetes (Class.)	Diabetes (Regr.)	CU (DNA)	CU (Kingdom)	MIMIC-IV
Baseline models	**Checkpoint averaging**	0.2117	0.75322	0.53356	0.06585	0.06378	0.1527
Normal ensemble models	**Checkpoint averaging**	0.2497	0.2790	0.5604	0.0007	0.2264	n/a
	**Random submodel**	0.1392	0.3062	0.5075	0.0440	0.01722	n/a
	**Submodel averaging**	0.1952	0.4597	0.4713	0.3882	0.1738	n/a
	**CA-SA**	0.2906	0.551	0.5193	0.5654	0.1638	n/a
Explanation ensemble models	**Checkpoint averaging**	0.2485	0.0175	0.5322	0.2510	0.2695	0.2954
	**Random submodel**	0.1365	0.2641	0.0625	0.2939	0.0193	0.01333
	**Submodel averaging**	0.2983	0.0355	0.8389	0.0953	0.0330	0.2080
	**CA-SA**	0.3964	0.89222	0.8529	0.6462	0.3481	0.1784

We verify this hypothesis further by analysing the results of the checkpoint-averaging-followed-by-submodel-averaging (CA-SA) experiments reported in Table 5. By analysing the results in the normal ensemble models we see that this combination of techniques increases the explanation consistency of the models, implying that averaging at both stages of the model is required. The results of the same experiment on explanation ensembles back this up, with our proposed architecture improving again upon the results of the normal ensemble CA-SA experiments. Thus, the benefits of explanation ensembles followed by CA-SA are two-fold: (1) it improves explanation consistency even further, and (2) it results in a much smaller model that can be run at inference time, significantly reducing computational costs whilst adding very little to the (one-time) training cost.

### Explanation ensemble size results

**Figure 4:**
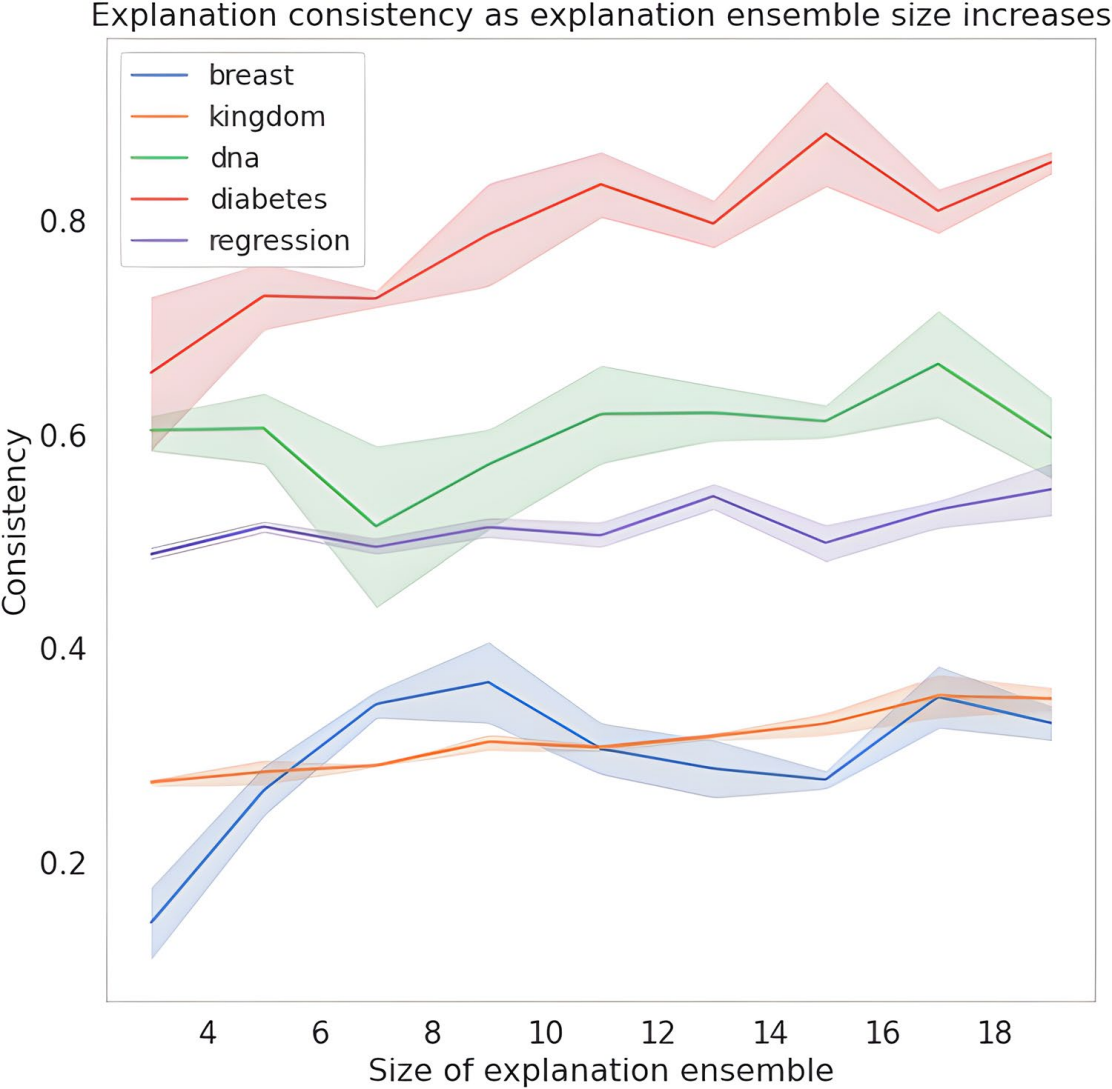
Explanation consistency (± standard error) of explanation ensembles as the number of sub-models within an ensemble increases across all datasets.

Research suggests that larger ensembles result in improved performance^10^. We find this also transfers to our explanation ensembles with Fig. 4 showing how, in general, explanation consistency increases as the number of sub-models increases. Intuitively, this is to be expected—the more sub-models present in an ensemble, the wider the range of parameters available for the ensemble to “average out” over.

It is interesting to consider why the point at which the consistency beings to plateau differs across datasets (and even different tasks with the same dataset. We hypothesise this is due to the number of features that are causally related to target versus how many features are spuriously correlated with the target. Explanation ensembles are designed such that the spurious correlations will be “averaged out” as the sub-models gradually learn to utilise only features present across all sub-models, and so in the ideal scenario the whole set of spurious features is covered by (at least) one of the explanation ensemble sub-models. Considering this hypothetical ideal scenario, it is clear that datasets with a smaller set of spurious features will require a smaller set of sub-models to achieve the best consistency by an explanation ensemble architecture possible. This hypothesis also extends to different tasks within the same dataset—each task will have a different subset of the dataset’s features, one of which will be smaller than the other. Although it is outside the scope of this introductory paper, it would be interesting for future work to consider this relationship in more detail.

### Explanation quality metrics

Tables 12 and 13 in the Supplementary Material report the explanation infidelity and sensitivity max^30^ on each individual baseline and explanation ensemble model tested. Across all datasets, each model has low explanation infidelity and sensitivity max—this confirms that SHAP is producing explanations that are faithful to the models. As the reported infidelity measure is the mean infidelity across the whole dataset, this also shows that the explanation methods provide global fidelity.

As Supplementary Table 13 shows, explanations generated from explanation ensembles are also high quality; the range and spread of the values is the same as the baseline models, implying that the new architecture does not affect the quality of the produced explanations. Importantly, this confirms that the explanations are also faithful to explanation ensembles, meaning that the improved explanation consistency is due to the changes in the architecture (i.e. the SHAP discriminator) rather than inconsistencies present in the explanation generation method (i.e. SHAP, in this case).

### Statistical significance results

We report both the test statistic *U* and the *p* value for both the performance metric and explanation separability comparisons between the baseline and explanation ensemble models. Figures 2c and 3 also show for which datasets we report statistically significant results. Table 6 reports the relevant values for each dataset.

**Table 6:** *U* test statistic and *p* values as calculated for the differences between the model performance and explanation separability $S_{(a, b)}$ of the baseline and explanation ensemble models; a two-sided test was used.

Dataset (Task)	Model performance		Explanation consistency	
	*U* Statistic	*p* value	*U* statistic	*p* value
BCW	75	0.00249292	774	0.009378
KAIMRC (Regression)	81	0.00040946	6475	$1.634 \times 10^{-6}$
KAIMRC (Classification)	51	0.04988344	3066	$6.382 \times 10^{-13}$
Codon usage (DNA)	81	0.00039825	5606.5	$3.855 \times 10^{-13}$
Codon usage (Kingdom)	0	0.00018267	11205	$1.179 \times 10^{-12}$
MIMIC-IV	72	0.10397974	1350	$8.226 \times 10^{-16}$

Across all datasets, the results of the Mann-Whitney tests support our conclusion that our proposed explanation ensemble architecture results in significantly improved explanation consistency *C*; all of the hypothesis tests result in significant results, highlighting that there is a significant difference between the results. This, coupled with the visualisation of explanation separability and JSD in Fig. 2b and c, provide strong evidence that our proposed technique significantly increases explanation consistency.

## Discussion

It is clear from both our initial consistency results on the baseline models, and from the corresponding studies carried out in^23^, that the inconsistency of explanations is an important issue that is present in across a range of deep learning models; we hypothesise that it is a direct result of the stochasticity of training. Recent reports from industry^42–44^ underline the importance of having explainable ML in industry (especially in sectors such as healthcare and finance), and how the lack of good quality explanations and the “unpredictable” nature of ML (which is highlighted by the inconsistency of explanations) are seen as barriers to wider adoption.

In this paper, we have presented an entirely new architecture that can be trained specifically to learn more consistently. Through thorough experimentation on tabular data, we have shown that both of these methods are able to produce significantly better explanations (in regards to their consistency) whilst still retaining high levels of model performance and explanation quality (as measured through other, non-consistency, quantities. Through the use of a wide range of tasks we have demonstrated that our proposed methods are able to work across both binary and multi-class classification, as well as regression, tasks and have exhibited the usefulness of our techniques in the healthcare sector by focusing on healthcare datasets.

Through experimentation with multiple different model weight averaging techniques, we have shown that checkpoint averaging followed by ensemble submodel averaging can improve explanation consistency. Through the application of this technique to our explanation ensemble architecture, we show that our architecture can beat the explanation consistency of current state of the art techniques even further whilst also significantly reducing the cost of running our proposed network at inference time. The final result is a comprehensive step towards creating consistent, robust models that can be deployed in sensitive domains such as healthcare and finance.

Future work should investigate the applicability of our proposed techniques to different data modalities such as images and text, along with the wide range of differing model architectures used for these different modalities. Furthermore, we encourage further improvements to explanation consistency to be explored: although our techniques see impressive improvements of up to 4 times the explanation consistency of the baseline models, the consistency for some of the datasets/tasks tested is still relatively low. This raises another question for future study: what properties of a dataset makes it easier for consistent (and, as an extension, causal) features to be learned. It would be interesting to explore the implications our technique has on the causality of the produced models—future work could investigate the degree to which causal features are learned by our model rather than correlated features.

## Supplementary Information

Supplementary Information.

## Data Availability

The Breast Cancer Wisconsin data is freely available: https://archive.ics.uci.edu/ml/datasets/breast+cancer+wisconsin+(diagnostic). The Codon Usage dataset is also publicly available: https://archive.ics.uci.edu/ml/datasets/Codon+usage. The KAIMRC dataset used is a third-party private clinical data that is not publicly available. The MIMIC-IV dataset is freely available to researchers after completing data privacy accreditation as set out by MIT, the data owner: https://mimic.mit.edu. This process is in place for privacy and legal purposes.
