# Author Correction: Using model explanations to guide deep learning models towards consistent explanations for EHR data

**DOI:** 10.1038/s41598-023-28610-3

**Published:** 2023-01-24

**Authors:** Matthew Watson, Bashar Awwad Shiekh Hasan, Noura Al Moubayed

**Affiliations:** 1 Department of Computer Science, Durham University, Durham, UK; 2 Evergreen Life Ltd, Manchester, UK

Correction to: *Scientific Reports* https://doi.org/10.1038/s41598-022-24356-6, published online 18 November 2022

The Acknowledgements section in the original version of this Article was incomplete.

“This work is supported by Grant 25R17P01847 from the European Regional Development Fund and Evergreen Life Ltd.”

now reads:

“This work is supported by Grant 25R17P01847 from the European Regional Development Fund and Evergreen Life Ltd. This work was supported by Innovate UK grant number 10027358.”

The original Article has been corrected.

