# Supplementary material for: Using model explanations to guide deep learning models towards consistent explanations for EHR data
Source: Sci Rep. 2022 Nov 18;12:19899. doi: 10.1038/s41598-022-24356-6 (PMC9674624; doi:10.1038/s41598-022-24356-6)
Supplement: Supplementary file 1 — Supplementary Information. [file 41598_2022_24356_MOESM1_ESM.pdf]

Using Model Explanations to Guide Deep Learning Models Towards Consistent Explanations for EHR Data

Matthew Watson
Department of Computer Science
Durham University
Durham, UK

Bashar Awwad Shiekh Hasan
Department of Computer Science
Durham University
Durham, UK

Noura Al Moubayed
Department of Computer Science
Durham University
Durham, UK

October 2022

**Dataset (Task)**	**Random Seed**	**Shuffle**	**Performance Metric**
Diabetes (Classification)	18829	FALSE	82.33
Diabetes (Classification)	20526	FALSE	82.93
Diabetes (Classification)	44392	FALSE	83.02
Diabetes (Classification)	7021	FALSE	82.71
Diabetes (Classification)	93864	FALSE	81.89
Diabetes (Classification)	17884	TRUE	83.08
Diabetes (Classification)	26549	TRUE	83.58
Diabetes (Classification)	42609	TRUE	83.53
Diabetes (Classification)	52732	TRUE	83.42
Diabetes (Classification)	53291	TRUE	83.33
Diabetes (Classification)	58075	TRUE	83.43
Diabetes (Classification)	65452	TRUE	83.27
Diabetes (Classification)	66701	TRUE	**83.67**
Diabetes (Classification)	7495	TRUE	83.33
Diabetes (Classification)	81189	TRUE	83.43
Diabetes (Classification)	9937	TRUE	83.12
Diabetes (Regression)	1	FALSE	0.578
Diabetes (Regression)	17351	FALSE	0.601
Diabetes (Regression)	35397	FALSE	0.579
Diabetes (Regression)	39419	FALSE	0.595
Diabetes (Regression)	42290	FALSE	0.601
Diabetes (Regression)	51363	FALSE	**0.602**
Diabetes (Regression)	54867	TRUE	0.582
Diabetes (Regression)	64500	TRUE	0.593
Diabetes (Regression)	66703	TRUE	0.569
Diabetes (Regression)	83349	TRUE	0.560
Diabetes (Regression)	95279	TRUE	0.584
Diabetes (Regression)	96047	TRUE	0.586

Table 1: Performance and hyperparameters of the baseline MLPs trained on the KAIMRC dataset. The performance metric for the classification task is accuracy and adjusted R^2 for the regression task.

**Dataset**	**Seed**	**Shuffle**	**ROC-AUC**
BCW	22323	FALSE	93.86
BCW	25197	FALSE	93.86
BCW	29698	FALSE	93.86
BCW	30135	FALSE	89.47
BCW	30938	FALSE	93.86
BCW	39325	FALSE	92.98
BCW	41292	FALSE	90.35
BCW	53050	FALSE	92.11
BCW	61455	FALSE	89.47
BCW	78827	FALSE	92.11
BCW	81960	FALSE	92.11
BCW	19191	TRUE	88.60
BCW	23087	TRUE	91.23
BCW	24735	TRUE	89.47
BCW	43842	TRUE	**95.61**
BCW	47506	TRUE	86.42
BCW	57075	TRUE	92.11
BCW	62605	TRUE	90.25
BCW	63612	TRUE	93.86
BCW	67425	TRUE	90.35
BCW	92747	TRUE	93.86
BCW	97704	TRUE	94.74

Table 2: Accuracy and hyperparameters for the baseline MLPs on the Breast Cancer Wisconsin (BCW) dataset.

Dataset (Task)	Seed	Shuffle	Accuracy	F1 Score
Codon Usage (DNA)	22402	FALSE	99.31	99.30
Codon Usage (DNA)	24402	FALSE	99.19	99.19
Codon Usage (DNA)	39126	FALSE	**99.38**	**99.39**
Codon Usage (DNA)	44437	FALSE	99.31	99.31
Codon Usage (DNA)	55833	FALSE	99.15	99.16
Codon Usage (DNA)	58236	TRUE	99.19	99.19
Codon Usage (DNA)	6160	TRUE	99.31	99.31
Codon Usage (DNA)	64119	TRUE	99.11	99.12
Codon Usage (DNA)	64390	TRUE	99.07	99.09
Codon Usage (DNA)	71650	TRUE	99.15	99.15
Codon Usage (Kingdom)	17094	FALSE	85.62	0.8504
Codon Usage (Kingdom)	19709	FALSE	**86.65**	**0.8638**
Codon Usage (Kingdom)	29559	FALSE	86.31	0.8616
Codon Usage (Kingdom)	3440	FALSE	85.67	0.8488
Codon Usage (Kingdom)	39406	FALSE	82.15	0.8103
Codon Usage (Kingdom)	51088	TRUE	83.30	0.8231
Codon Usage (Kingdom)	63023	TRUE	85.84	0.8506
Codon Usage (Kingdom)	74147	TRUE	85.79	0.8547
Codon Usage (Kingdom)	84013	TRUE	85.75	0.8530
Codon Usage (Kingdom)	92214	TRUE	86.22	0.8580

Table 3: Accuracy, F1 score and hyperparameters of the baseline MLPs trained on the Codon Usage dataset on both the kingdom and DNA multi-class classification tasks.

**Dataset**	**Seed**	**Shuffle**	**AUROC**
MIMIC-IV	7321	FALSE	0.7642
MIMIC-IV	1163	FALSE	0.7247
MIMIC-IV	3193	FALSE	0.7813
MIMIC-IV	7429	FALSE	0.7563
MIMIC-IV	8433	FALSE	0.7916
MIMIC-IV	22321	TRUE	**0.8166**
MIMIC-IV	32283	TRUE	0.7748
MIMIC-IV	69432	TRUE	0.7794
MIMIC-IV	77973	TRUE	0.8071
MIMIC-IV	82342	TRUE	0.8089

Table 4: Model performance and hyperparameters for the LSTM-based baseline models trained on MIMIC-IV mortality prediction.

Dataset (Task)	Seed	Shuffle	Accuracy
BCW	1621	FALSE	72.42
BCW	3063	FALSE	84.62
BCW	3309	FALSE	87.25
BCW	7159	FALSE	78.57
BCW	8163	FALSE	86.59
BCW	2602	TRUE	72.64
BCW	3233	TRUE	**88.79**
BCW	6922	TRUE	88.24
BCW	7797	TRUE	76.15
BCW	8332	TRUE	72.86

Table 5: Model performance of normal ensemble models, of 10 sub-models each, on the BCW dataset.

Dataset (Task)	Seed	Shuffle	Accuracy
KAIMRC (Classification)	1621	FALSE	83.28
KAIMRC (Classification)	3063	FALSE	83.15
KAIMRC (Classification)	3309	FALSE	83.07
KAIMRC (Classification)	7159	FALSE	**83.64**
KAIMRC (Classification)	8163	FALSE	83.08
KAIMRC (Classification)	2602	TRUE	83.15
KAIMRC (Classification)	3233	TRUE	83.08
KAIMRC (Classification)	6922	TRUE	83.28
KAIMRC (Classification)	7797	TRUE	83.28
KAIMRC (Classification)	8332	TRUE	83.27
KAIMRC (Regression)	1621	FALSE	0.52
KAIMRC (Regression)	3063	FALSE	0.49
KAIMRC (Regression)	3309	FALSE	**0.54**
KAIMRC (Regression)	7159	FALSE	0.51
KAIMRC (Regression)	8163	FALSE	0.51
KAIMRC (Regression)	2602	TRUE	0.51
KAIMRC (Regression)	3233	TRUE	0.52
KAIMRC (Regression)	6922	TRUE	0.50
KAIMRC (Regression)	7797	TRUE	0.53
KAIMRC (Regression)	8332	TRUE	0.51

Table 6: Model performance of normal ensemble models, of 10 sub-models each, on the KAIMRC dataset for both the classification and regression tasks.

**Dataset (Task)**	**Seed**	**Shuffle**	**Accuracy**
Codon Usage (DNA)	1621	FALSE	99.07
Codon Usage (DNA)	3063	FALSE	99.42
Codon Usage (DNA)	3309	FALSE	99.23
Codon Usage (DNA)	7159	FALSE	98.84
Codon Usage (DNA)	8163	FALSE	99.19
Codon Usage (DNA)	2602	TRUE	**99.46**
Codon Usage (DNA)	3233	TRUE	99.00
Codon Usage (DNA)	6922	TRUE	99.04
Codon Usage (DNA)	7797	TRUE	98.88
Codon Usage (DNA)	8332	TRUE	99.23
Codon Usage (Kingdom)	1621	FALSE	90.39
Codon Usage (Kingdom)	3063	FALSE	87.23
Codon Usage (Kingdom)	3309	FALSE	90.82
Codon Usage (Kingdom)	7159	FALSE	91.24
Codon Usage (Kingdom)	8163	FALSE	90.99
Codon Usage (Kingdom)	2602	TRUE	91.20
Codon Usage (Kingdom)	3233	TRUE	91.29
Codon Usage (Kingdom)	6922	TRUE	88.63
Codon Usage (Kingdom)	7797	TRUE	89.96
Codon Usage (Kingdom)	8332	TRUE	**91.76**

Table 7: Model performance of normal ensemble models, of 10 sub-models each, on the Codon Usage dataset for both the DNA and kingdom multi-class classification tasks.

**Dataset (Task)**	**Seed**	**Shuffle**	**Accuracy**
BCW	15671	FALSE	80.59
BCW	19353	FALSE	89.47
BCW	26628	FALSE	**90.53**
BCW	45386	FALSE	88.90
BCW	56945	FALSE	89.63
BCW	58245	TRUE	89.24
BCW	59288	TRUE	86.84
BCW	92627	TRUE	83.33
BCW	99734	TRUE	87.72

Table 8: Model performance of our explanation ensemble models, each of 10 sub-models each, on the BCW dataset.

**Dataset (Task)**	**Seed**	**Shuffle**	**Accuracy**
KAIMRC (Classification)	3294	FALSE	81.86
KAIMRC (Classification)	32259	FALSE	81.82
KAIMRC (Classification)	45556	FALSE	82.37
KAIMRC (Classification)	56208	FALSE	82.64
KAIMRC (Classification)	61300	TRUE	81.61
KAIMRC (Classification)	78867	TRUE	**83.27**
KAIMRC (Classification)	80154	TRUE	82.28
KAIMRC (Classification)	83464	TRUE	82.53
KAIMRC (Regression)	1540	FALSE	0.5493
KAIMRC (Regression)	4881	FALSE	0.5152
KAIMRC (Regression)	33097	FALSE	0.5514
KAIMRC (Regression)	43716	FALSE	0.5529
KAIMRC (Regression)	45016	TRUE	0.5254
KAIMRC (Regression)	62778	TRUE	0.5572
KAIMRC (Regression)	72795	TRUE	0.5561
KAIMRC (Regression)	91774	TRUE	0.5076
KAIMRC (Regression)	97880	TRUE	**0.5578**

Table 9: Model performance of our explanation ensemble models, each of 10 submodels each, on the KAIMRC dataset for both the classification and regression tasks.

**Dataset (Task)**	**Seed**	**Shuffle**	**Accuracy**
Codon Usage (DNA)	7009	FALSE	**98.51**
Codon Usage (DNA)	20624	FALSE	98.26
Codon Usage (DNA)	37971	FALSE	98.49
Codon Usage (DNA)	41030	FALSE	97.51
Codon Usage (DNA)	43356	FALSE	97.39
Codon Usage (DNA)	64863	TRUE	97.06
Codon Usage (DNA)	86245	TRUE	97.64
Codon Usage (DNA)	94742	TRUE	98.37
Codon Usage (DNA)	97499	TRUE	97.54
Codon Usage (Kingdom)	2107	FALSE	89.96
Codon Usage (Kingdom)	46598	FALSE	87.12
Codon Usage (Kingdom)	47329	FALSE	89.87
Codon Usage (Kingdom)	49806	FALSE	89.27
Codon Usage (Kingdom)	49951	FALSE	89.66
Codon Usage (Kingdom)	54426	TRUE	87.64
Codon Usage (Kingdom)	57058	TRUE	88.15
Codon Usage (Kingdom)	64179	TRUE	**90.26**
Codon Usage (Kingdom)	73122	TRUE	88.80
Codon Usage (Kingdom)	87606	TRUE	89.48

Table 10: Model performance of our explanation ensemble models, with of 10 sub-models each, on the Codon Usage dataset on both the DNA and Kingdom multi-class classification tasks.

**Dataset (Task)**	**Seed**	**Shuffle**	**AUROC**
MIMIC-IV	1163	FALSE	0.7733
MIMIC-IV	22321	FALSE	0.7734
MIMIC-IV	3193	FALSE	0.7734
MIMIC-IV	32283	FALSE	**0.7735**
MIMIC-IV	69432	FALSE	0.7733
MIMIC-IV	7321	TRUE	0.7730
MIMIC-IV	7429	TRUE	0.7732
MIMIC-IV	77973	TRUE	0.7733
MIMIC-IV	82342	TRUE	0.7732
MIMIC-IV	8433	TRUE	0.7733

Table 11: Model performance (accuracy under the receiver operating characteristic curve, AUROC) of our explanation ensemble models, of 10 sub-models each, on the MIMIC-IV mortality prediction task.

**Dataset**	**Seed**	**Shuffle**	**Infidelity**	**Sensitivity**
BCW	29698	FALSE	0.000391	0.581461
BCW	25197	FALSE	0.000634	0.512061
BCW	41292	FALSE	0.000527	0.421115
BCW	22323	FALSE	0.000434	0.517406
BCW	19191	TRUE	0.000445	0.513631
BCW	24735	TRUE	0.000460	0.563020
BCW	47506	TRUE	0.000774	0.716147
BCW	57075	TRUE	0.001268	0.617298
Codon Usage (DNA)	71650	TRUE	0.008231	0.678376
Codon Usage (DNA)	58236	TRUE	0.001719	1.823636
Codon Usage (DNA)	64119	TRUE	0.003357	1.089292
Codon Usage (DNA)	64390	TRUE	0.004377	1.255262
Codon Usage (DNA)	22402	FALSE	0.001696	1.610876
Codon Usage (DNA)	6160	TRUE	0.002804	1.356076
Codon Usage (DNA)	39126	FALSE	0.000410	3.270923
Codon Usage (DNA)	55833	FALSE	0.005762	0.776691
Codon Usage (DNA)	44437	FALSE	0.005224	0.997720
KAIMRC (Classification)	1621	FALSE	0.001360	0.591762
KAIMRC (Classification)	3063	FALSE	0.004201	0.453467
KAIMRC (Classification)	3309	FALSE	0.001295	0.509114
KAIMRC (Classification)	7159	FALSE	0.000000	0.000000
KAIMRC (Classification)	8163	FALSE	0.003186	0.427264
KAIMRC (Classification)	2602	TRUE	0.003995	0.397451
KAIMRC (Classification)	3233	TRUE	0.001077	0.542822
KAIMRC (Classification)	6922	TRUE	0.000547	0.000000
KAIMRC (Classification)	7797	TRUE	0.003540	0.597366
KAIMRC (Classification)	8332	TRUE	0.000997	0.527902
Codon Usage (Kingdom)	1621	FALSE	0.002073	0.700954
Codon Usage (Kingdom)	3063	FALSE	0.006445	0.968512
Codon Usage (Kingdom)	7159	FALSE	0.009926	0.754319
Codon Usage (Kingdom)	8163	FALSE	0.004835	0.688471
Codon Usage (Kingdom)	2602	TRUE	0.002424	0.673015
Codon Usage (Kingdom)	3233	TRUE	0.039926	0.573023
Codon Usage (Kingdom)	6922	TRUE	0.012041	0.718585
Codon Usage (Kingdom)	7797	TRUE	0.003896	1.015589

Table 12: Explanation infidelity and explanation sensitivity max of the baseline model architectures on all classification datasets.

**Dataset**	**Seed**	**Shuffle**	**Infidelity**	**Sensitivity**
BCW	1621	FALSE	0.000039834	1.114579
BCW	3063	FALSE	0.000075637	0.835891
BCW	3309	FALSE	0.000071457	1.704849
BCW	7159	FALSE	0.000081874	**0.633781**
BCW	8163	FALSE	0.000085646	0.998697
BCW	2602	TRUE	0.000022444	**0.633781**
BCW	3233	TRUE	0.000422726	0.995961
BCW	6922	TRUE	0.000442698	0.738602
BCW	7797	TRUE	**0.000010963**	1.175704
BCW	8332	TRUE	0.001441245	0.894858
Codon Usage (DNA)	1621	FALSE	0.00062983	**0.656578**
Codon Usage (DNA)	3063	FALSE	**0.00000066**	1.467080
Codon Usage (DNA)	3309	FALSE	0.00000144	0.739519
Codon Usage (DNA)	7159	FALSE	0.00006802	1.894022
Codon Usage (DNA)	8163	FALSE	0.00000257	0.780092
Codon Usage (DNA)	2602	TRUE	0.00001721	1.000341
Codon Usage (DNA)	3233	TRUE	0.00003930	1.979980
Codon Usage (DNA)	6922	TRUE	0.00000357	1.071276
Codon Usage (DNA)	7797	TRUE	0.00001502	0.761258
Codon Usage (DNA)	8332	TRUE	0.00144648	2.045398
Codon Usage (Kingdom)	1621	FALSE	0.00002163	0.930669
Codon Usage (Kingdom)	3063	FALSE	0.00003154	1.205581
Codon Usage (Kingdom)	3309	FALSE	0.00002493	0.951259
Codon Usage (Kingdom)	7159	FALSE	0.00003060	0.981180
Codon Usage (Kingdom)	8163	FALSE	**0.00001940**	1.083869
Codon Usage (Kingdom)	2602	TRUE	0.00003436	**0.789647**
Codon Usage (Kingdom)	3233	TRUE	0.00004075	1.104336
Codon Usage (Kingdom)	6922	TRUE	0.00003798	1.003424
Codon Usage (Kingdom)	7797	TRUE	0.00003327	1.121576
Codon Usage (Kingdom)	8332	TRUE	0.00002583	0.981720
KAIMRC (Classification)	1621	FALSE	0.00045833	0.483609
KAIMRC (Classification)	3063	FALSE	0.00090531	**0.464702**
KAIMRC (Classification)	3309	FALSE	**0.00024935**	0.484267
KAIMRC (Classification)	7159	FALSE	0.00043942	0.538328
KAIMRC (Classification)	8163	FALSE	0.00220850	0.471807
KAIMRC (Classification)	2602	TRUE	0.00334645	0.474118
KAIMRC (Classification)	3233	TRUE	0.00046485	0.563551
KAIMRC (Classification)	6922	TRUE	0.00100100	0.472233
KAIMRC (Classification)	7797	TRUE	0.00141688	0.516746
KAIMRC (Classification)	8332	TRUE	0.00079866	0.581355

Table 13: Explanation infidelity and explanation sensitivity of each individual explanation ensemble (of size 10) tested across each classification dataset.
